# Landscape heterogeneity in landform and land use provides functional resistance to gene flow in continuous Asian black bear populations

**DOI:** 10.1002/ece3.5102

**Published:** 2019-04-05

**Authors:** Naoki Ohnishi, Takeshi Osawa, Toshiaki Yamamoto, Reina Uno

**Affiliations:** ^1^ Tohoku Research Center Forestry and Forest Products Research Institute Morioka Japan; ^2^ Graduate School of Urban Environmental Sciences Tokyo Metropolitan University Hachiouji Tokyo Japan; ^3^ Department of Veterinary Nursing and Technology Nippon Veterinary and Life Science University Musashino Tokyo Japan; ^4^ Institute for Advanced Biosciences Keio University Tsuruoka Yamagata Japan

**Keywords:** barrier, genetic structure, land use, Landscape genetics, resistance, *Ursus thibetanus*

## Abstract

**Context:**

Genetic diversity is one of the most important facets of biological diversity, and changes in the spatial pattern of habitats, often modified by human activity, are believed to have affected the genetic diversity of resident natural populations.

**Objectives:**

We undertook a landscape genetic analysis in order to determine which landscape features influence gene flow within Asian black bear populations and to identify the underlying processes.

**Methods:**

In our evaluation of gene flow, we estimated four parameters of resistance with regard to landscape elevation: the mean, the difference between the highest and lowest, the standard deviation, and the coefficient of variation of elevation among individuals. We then examined the resistance effect of different land use types.

**Results:**

With the exception of mean elevation, we found that all parameters showed a significant relationship with genetic distance, indicating that unevenness in elevation provides functional resistance to gene flow. Although we found no evidence of landscape barriers (isolation‐by‐barrier), there was an indication of landscape resistance (isolation‐by‐resistance). Urban area and farmland are suggested to be the strong factors contributing to the resistance to gene flow, even though isolation‐by‐distance was also detected. When we examined gene flow for pairs of males and pairs of females, both isolation‐by‐distance and isolation‐by‐resistance were stronger in order of female pairs, male pairs, all individual pairs.

**Conclusions:**

We conclude that landscape resistance was detectable with a high contrast in landscape heterogeneity and they are more influential on females than males.

**OPEN PRACTICES:**

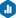

This article has been awarded Open Data badge. All materials and data are publicly accessible via the Open Science Framework at https://doi.org/10.5061/dryad.gn0qf16. Learn more about the Open Practices badges from the Center for Open Science: https://osf.io/tvyxz/wiki.

## 1 INTRODUCTION

Genetic diversity is one of the most important elements of biological diversity because it determines the fitness and survival of individuals, the viability of populations, and the ability of species to adapt to environmental change (Balkenhol, Cushman, Storfer, & Waits, 2016). The spatial pattern of habitats influences organism perception and behavior, which drive the higher‐level processes of population dynamics, gene flow, and adaptive evolution (Cushman, McRae, & McGarigal, 2016). However, human activity has, to varying degrees, modified the spatial pattern of habitats, which is believed to have affected the genetic diversity of resident natural populations.

Although many population genetic studies over the past few decades have revealed patterns in the genetic variation in mammal populations, few have statistically examined the underlying processes (Cushman, Shirk, & Landguth, 2013).

Population boundaries of the American black bear (*Ursus americanus*), which is known as a wide‐ranging omnivore that is dependent on forest habitat, are genetically obscure when the forest habitat is contiguous and the species is widely distributed. In the Great Lakes region in Canada, clinal structuring of distribution is induced by isolation‐by‐distance (IBD) at 550 km and anthropogenic effects are rarely observed (Pelletier et al., 2012). In contrast, the findings of two studies have indicated that landscape features have a greater influence on the genetic structure of black bear populations in mountains on the border between the states of Montana and Idaho than does Euclidean distance (Cushman, McKelvey, Hayden, & Schwartz, 2006; Short Bull et al. 2011). On the basis of an examination of the effect of land use, no landscape barriers (IBB: isolation‐by‐barrier) were detected, whereas there was an indication of landscape resistance (IBR: isolation‐by‐resistance). Moreover, resistance due to elevation has been suggested to be lowest in the midelevation range (Cushman et al., 2006). The authors of a further study concluded that the effect of resistance was more detectable in landscapes with high heterogeneity in elevation and land use (Short Bull et al. 2011).

Simulation studies have clarified that a higher contrast in landscape resistance between habitat and nonhabitat increases the ability to detect the influence of landscape resistance on gene flow (Cushman et al., 2013; Jaquiery, Broquet, Hirzel, Yearsley, & Perrin, 2011). It has also been suggested that genetic differentiation is independent of Euclidean distance and significantly related to landscape structure when habitats are highly fragmented (Cushman et al., 2013). It has, however, been argued that it is difficult to identify the landscape features that influence genetic variation without reference to Euclidean distance, because in most real landscapes, the landscape features are generally aggregated into a few patches.

**Figure 1:**
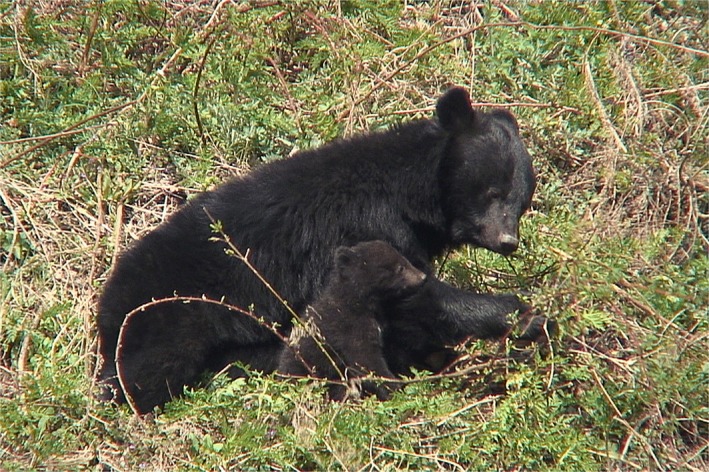
An Asian black bear cub with its mother in Iwate Prefecture, Japan. Photo by Y. Sato

Genetic variations in populations of the Asian black bear (*Ursus thibetanus*: Figure 1) have been well studied, and in Japan, genetic differences have been observed not only among fragmented populations but also in continuous populations (Ishibashi et al., 2016; Ishibashi & Saitoh, 2004; Ohnishi, Saitoh, Ishibashi, & Oi, 2007; Ohnishi, Uno, Ishibashi, Tamate, & Oi, 2009; Saitoh, Ishibashi, Kanamori, & Kitahara, 2001; Uno, Doko, Ohnishi, & Tamate, 2015; Yamamoto et al., 2012; Yasukochi, Kurosaki, Yoneda, & Koike, 2010; Yasukochi et al., 2009). Using a landscape ecology approach, it has been suggested that the present genetic structure reflects the mixed contribution of remnants of the ancestral population structure and current gene flow affected by human activities (Uno et al., 2015).

In the present study, we undertook a landscape genetic analysis in order to determine which landscape features influence gene flow within Asian black bear populations in the northern part of Japan and to identify the underlying processes. We considered that northern Japan would be a suitable area to examine these issues because it is characterized by a range of different topographical features and has a mix of both artificial and natural landscapes. The present study was conducted in three stages. Initially, we determined the genetic structure of continuous populations of the black bear using Bayesian clustering analysis. We then proceeded to assess gene flow resistance in terms of four parameters of elevation: the mean, the difference, the standard deviation, and the coefficient of variation of elevation among individuals. Finally, we examined the resistance effect of land use by comparing IBD, IBB, and IBR. We conducted these analyses for all sample pairs, pairs of males, and pairs of females. Due to male‐biased dispersal, we predicted that the relationships between genetic distance and each parameter would be detected in the following order, female pairs >all sample pairs >male pairs.

## 2 MATERIALS AND METHODS

### 2.1 DNA samples

DNA samples were obtained from a total of 148 individuals from the northern margins of a large continuous population of black bears in Japan. Among these, 44 bears in Aomori Prefecture were identified from bear hairs collected by Yamamoto et al. using noninvasive hair‐trapping (Yamamoto et al., 2012). A further 100 tissue samples were obtained in four other prefectures, and four samples were collected from bears in the east of Aomori Prefecture that had been hunted or captured for pest control between 1991 and 2011.

### 2.2 DNA analysis

Genomic DNA was extracted from tissue and hair samples using MagExtractor (TOYOBO) and an ISOHAIR kit (Nippongene Inc.), respectively. The genotypes at 16 microsatellite loci (G1A, G1D, G10B, G10J, G10O, G10L, G10M, G10P, G10X, MSUT‐1, MSUT‐2, MSUT‐6, MSUT‐7, UarMU05, UarMU23, and UarMU50) were determined for all bears by polymerase chain reactions (Kitahara, Isagi, Ishibashi, & Saitoh, 2000; Paetkau, Calvert, Stirlin, & Strobeck, 1995; Paetkau, Shields, & Strobeck, 1998; Taberlet et al., 1997).

### 2.3 Bayesian clustering and genetic statistics

The genotype data were analyzed using STRUCTURE Bayesian clustering software (Pritchard, Stephens & Donnelly, 2000) to determine the genetic structure of the black bear population. Initially, we calculated P_ID_ 10 times for each K at 1–10 with 5,000,000 Markov chain Monte Carlo (MCMC) iterations, with a burn‐in of 100,000, and independent allele frequencies (lambda 1.0), using an admixture model (alpha inferred, with initial alpha set to 1.0). The deltaK had a single peak when *K* = 2, thus we set *K* to 2, and assumed population inferred proportions using 10,000,000 MCMC iterations, with a burn‐in of 1,000,000.

After the Bayesian clustering, we estimated genetic statistics of each assumed population. Departure of the observed heterozygosity from the Hardy–Weinberg equilibrium (HWE) and linkage disequilibrium (LD) were examined using the Markov chain method with 10,000 permutations (Guo & Thompson, 1992) by using Arlequin 3.5.2.2 (Excoffier, Laval, & Schneider, 2005). The sequential Bonferroni method was used to adjust significance values for all multiple comparisons (Rice, 1989). The presence of null alleles and genotyping errors for each locus and each assumed population were examined using MicroChecker 2.2.3 (Van Oosterhout, 2004).

### 2.4 Analytical unit

We used ~1‐km grid cells for spatial analysis. This is one of the standard units used in Japan for gathering statistics relating to populations, land use, and wildlife. For analysis, we demarcated the sites of sample collection in grids.

### 2.5 The influence of landform on gene flow

We used Mantel tests to investigate the relationship between genetic distance and elevation parameters for all pairs of individuals. We constructed distance networks by initially connecting two points with a straight line for all individual pairs, that is, male–female pairs, all male pairs, and all female pairs. If the two points were located within the same 1‐km grid, Euclidean distance was defined as 0. If the two points were located in neighboring grids, Euclidean distance was approximated as 1 km according to the analytical unit. For each network line, we then determined four elevation‐related parameters: (a) the mean elevation within each network; (b) the difference between the highest and lowest elevation in each network; and (c) the standard deviation (*SD*) and (d) coefficient of variance (CV) of elevation within each network. The elevation variables for each network line were obtained from a 200‐m digital elevation model based on a 250‐m numerical map. Thereafter, we calculated Bray–Curtis percentage dissimilarity as the genetic distance between individuals using the ECODIST package in R ver. 2.14 (Goslee & Urban, 2007). Finally, Mantel tests (Manly, 1997) for genetic distance and candidate elevation parameters were performed with 999 permutations using the vegan 2.5‐1 package in R.

### 2.6 The influence of land use on gene flow

We examined which landscape features have the potential to function as barriers or resistance to gene flow among individuals. For this analysis, we calculated the amounts of different land use types within the network lines. To this end, we used seven land use types (forest, grass field, farmland, urban area, open water, special matrix, and wetland) using the 1‐km cell grids of a land use classification map (Ogawa et al., 2013). The special matrix includes natural bare ground, limestone vegetation, volcanic desert, solfatara desert, and cliffs. We collated the data on land use for all network lines and calculated the number of classes in each network. In total, we assessed 138.4 billion grids for all networks (Table 1). Among these, forest represented the largest (64.6%) area, followed by grass field (20.3%). Special matrix, which comprises areas such as cliffs and bare ground, occurred in 72 million grids (0.052%).

**Table 1:** The number of grids of land use types in the study area

Land use	No. grids
Forest	89,458,000,000
Grass field	28,120,000,000
Farmland	17,834,000,000
Urban area	2,170,000,000
Open water	722,000,000
Special matrix	72,000,000
Wetland	25,000,000

Note

Special matrix means natural bare ground, limestone vegetation, volcanic desert, solfatara desert, and cliffs.

We investigated the resistance effect of land use on gene flow by using simple and partial Mantel tests according to the concept of Ruiz‐Gonzalez et al. (2014). Initially, we conducted a simple Mantel test (*G × R_L_*) for genetic distance and the candidate land use class with 999 permutations. In this analysis, we calculated the Mantel *r* value between genetic distance and the resistance value of each candidate land use class. Here, resistance values were obtained for the product of the number of grids of the candidate land use class within the network and presumed resistance (2, 5, 25, 50, and 100). Assigning resistance values to landscape features is a well‐known challenge in landscape genetics (Tucker, Allendorf, Truex, & Schwartz, 2017), and thus, we assigned each landscape feature with a wide range of resistance values. Next, we performed partial Mantel tests between genetic distance and the resistance value (2, 5, 25, 50, and 100) of the candidate land use with partialling out of Euclidean distance of the network (*G × R_L_|Dis*) and between genetic distance and Euclidean distance with partialling out the resistance value of candidate land use (*G × Dis|R_L_*). When *G × R_L_|Dis* was statistically significant (*p* < 0.05) and *G × Dis|R_L_* was negative or not statistically significant (*p* > 0.05) in these partial Mantel tests, we regarded that the candidate land use influenced gene flow via resistance according to Ruiz‐Gonzalez et al. (2014). Subsequent to these tests, we additionally estimated the correlation between genetic distance and the combined resistance of land uses by using simple Mantel tests (*G* × Σ*R_L_*).

We also performed simple and partial Mantel tests to determine whether different land uses function as barriers to gene flow with the same resistance effect. We examined the correlation between genetic distance and the presence/absence of a candidate land use (0 or 1) using a simple Mantel test (*G × B_L_*). Partial Mantel tests were used to assess the relationship between genetic distance and the presence of candidate land use with partialling out of Euclidean distance of the network (*G × B_L_|Dis*) and between genetic distance and Euclidean distance with partialling out of the presence of a candidate land use (*G × Dis|B_L_*). When *G × B_L_|Dis* was statistically significant (*p* < 0.05) and *G × Dis|B_L_* was negative or not statistically significant (*p* > 0.05) in these partial Mantel tests, we regarded the candidate land use as representing a functional barrier to gene flow according to Ruiz‐Gonzalez et al. (2014).

## 3 RESULTS

### 3.1 Genetic structure

**Figure 2:**
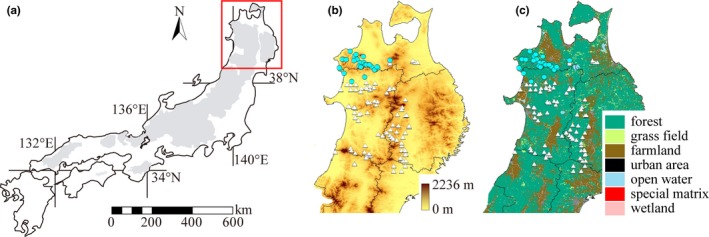
Maps of the study area and relevant adjacent regions: light shadings show the distribution of the Asian black bear (*Ursus thibetanus*) in Japan (a); colored maps represent elevation (b) and land use (c) of the study area; sites where bears were captured (b and c); and the different symbols indicate the location of genetic clusters inferred using the Bayesian clustering software STRUCTURE

Bayesian clustering analysis inferred five genetic clusters from the 148 genotyped samples. Among these, 126 samples had a high assignment ratio (>0.8) to one cluster when *K* = 2, and a spatial structuring of clusters was observed (Figure 2). After the Bayesian clustering analysis, we checked the validity of markers for each assumed population with *K* = 2. Null alleles were indicated at two loci (G10B and MSUT‐7) in larger assumed population, but neither LD nor HWE deficiency was observed in both assumed population.

### 3.2 The influence of landform on gene flow

The results of Mantel tests, which were used to estimate the relationship between the genetic distance and elevation parameters of all individual networks, are shown in Table 2. We found that the mean elevation within each network was not significantly related to the genetic distance for all individuals, males, and females. In contrast, the difference between the highest and lowest elevation in each network, and the *SD* and CV within each network all showed higher Mantel *r* values than the null model (the Euclidean distance) for all three pair categories and male pairs. CV had highest Mantel *r* values for all three network categories, and only CV showed higher Mantel *r* values than the Euclidean distance in female pairs. For all parameters, females had larger Mantel *r* values than males.

**Table 2:** Results of landscape resistance of landform

Model of elevation	All samples (*n* = 148)		Males (*n* = 87)		Females (*n* = 35)	
	Mantel *r*	*p* value	Mantel *r*	*p* value	Mantel *r*	*p* value
Null	0.0687	0.001	0.1388	0.001	0.2373	0.001
Mean	−0.0701	0.993	−0.0267	0.798	0.0490	0.187
Max. difference of elevation	0.1258*	0.001	0.1852*	0.001	0.2373	0.001
Standard deviation	0.1205*	0.001	0.1692*	0.001	0.1954	0.001
Coefficient of variance	0.2087*	0.001	0.2678*	0.001	0.3511*	0.001

Note

Asterisks indicate that Mantel *r* values are higher than those of null model (the Euclidean distance).

### 3.3 The resistance function of land use on gene flow

We performed simple Mantel tests to determine the relationship between genetic distance and each resistance value (2, 5, 25, 50, and 100) of land use types (*G × R_L_*,), and found that for all sample pairs, farmland, and urban area showed higher Mantel *r* values for all resistance values than the null model (the Euclidean distance: Mantel *r* = 0.0687, Table 3). The resistance indicating the highest Mantel *r* was 25 for both land use types (Mantel *r* = 0.0779 for farmland and 0.0721 for urban area). Next, we performed partial Mantel tests with partialling out of Euclidean distance of the network (*G × R_L_|Dis*) and between genetic distance and Euclidean distance with partialling out the resistance value of the candidate land use (*G × Dis|R_L_*). For farmland and urban areas, we found that the Mantel *r* values for *G × R_L_|Dis* were positively significant (*p* < 0.05), whereas the Mantel *r* values for *G × Dis|R_L_* were statistically nonsignificant (*p* > 0.05), with the exception of resistance values 2 and 5 for urban area. Thus, according to the concept proposed by Ruiz‐Gonzalez et al. (2014), both farmland and urban area have a significant resistance influence on genetic distance for all sample pairs.

**Table 3:** Results of simple and partial Mantel test detecting landscape resistance of land use

Model of land use	Resistance	G*RL		G*RL|Dis		G*Dis|RL	
		Mantel *r*	*p* value	Mantel *r*	*p* value	Mantel *r*	*p* value
All samples (*n* = 148)							
Null		0.0687	0.001				
Forest	2	0.0664	0.002	−0.0116	0.749	0.0211	0.093
	5	0.0658	0.001	−0.0033	0.607	0.0201	0.133
	25	0.0663	0.001	0.0021	0.463	0.0183	0.166
	50	0.0663	0.001	0.0024	0.450	0.0182	0.145
	100	0.0663	0.001	0.0024	0.445	0.0182	0.162
Grass field	2	0.0672	0.001	−0.0220	0.950	0.0263	0.014
	5	0.0644	0.001	−0.0196	0.944	0.0309	0.006
	25	0.0565	0.003	−0.0247	0.969	0.0463	0.001
	50	0.0546	0.005	−0.0263	0.975	0.0494	0.001
	100	0.0539	0.002	−0.0272	0.985	0.0506	0.001
Farmland	2	0.0728*	*0.001*	*0.0315*	*0.033*	*−0.0204*	*0.885*
	5	0.0777*	*0.001*	*0.0380*	*0.020*	*−0.0110*	*0.754*
	25	0.0779*	***0.001***	*0.0374*	*0.015*	*0.0069*	*0.336*
	50	0.0772*	*0.001*	*0.0364*	*0.018*	*0.0093*	*0.298*
	100	0.0770*	*0.001*	*0.0361*	*0.023*	*0.0097*	*0.296*
Urban area	2	0.0693*	0.001	0.0140	0.121	−0.0106	0.802
	5	0.0707*	0.001	0.0201	0.060	−0.0113	0.822
	25	0.0721*	***0.001***	*0.0225*	*0.046*	*−0.0048*	*0.678*
	50	0.0720*	*0.002*	*0.0216*	*0.044*	*−0.0022*	*0.593*
	100	0.0717*	*0.001*	*0.0205*	*0.042*	*−0.0006*	*0.513*
Open water	2	0.0681	0.001	−0.0171	0.931	0.0193	0.038
	5	0.0669	0.001	−0.0171	0.907	0.0231	0.019
	25	0.0640	0.001	−0.0198	0.958	0.0320	0.005
	50	0.0631	0.001	−0.0208	0.954	0.0343	0.004
	100	0.0627	0.001	−0.0215	0.956	0.0354	0.003
Special matrix	2	0.0686	0.001	−0.0088	0.821	0.0097	0.185
	5	0.0684	0.001	−0.0087	0.782	0.0109	0.144
	25	0.0679	0.001	−0.0097	0.815	0.0145	0.090
	50	0.0677	0.001	−0.0103	0.832	0.0157	0.068
	100	0.0676	0.001	−0.0107	0.846	0.0163	0.069
Wetland	2	0.0687	0.001	−0.0070	0.751	0.0072	0.233
	5	0.0686	0.001	−0.0064	0.706	0.0072	0.237
	25	0.0685	0.001	−0.0062	0.747	0.0081	0.224
	50	0.0685	0.001	−0.0062	0.702	0.0084	0.213
	100	0.0684	0.001	−0.0064	0.729	0.0087	0.190
Males (*n* = 87)							
Null		0.1388	0.001				
Forest	2	0.1364	0.001	−0.0066	0.627	0.0268	0.120
	5	0.1382	0.001	0.0162	0.248	0.0204	0.180
	25	0.1397*	**0.001**	0.0262	0.115	0.0206	0.191
	50	0.1396*	0.001	0.0261	0.132	0.0212	0.189
	100	0.1396*	0.001	0.0261	0.114	0.0212	0.195
Grass field	2	0.1367	0.001	−0.0275	0.924	0.0364	0.029
	5	0.1326	0.001	−0.0241	0.887	0.0477	0.006
	25	0.1235	0.001	−0.0215	0.863	0.0673	0.001
	50	0.1206	0.001	−0.0243	0.889	0.0733	0.001
	100	0.1198	0.001	−0.0255	0.908	0.0751	0.001
Farmland	2	0.1429*	0.001	0.0374	0.068	−0.0144	0.735
	5	0.1467*	*0.001*	*0.0483*	*0.021*	*0.0059*	*0.365*
	25	0.1485*	***0.001***	*0.0604*	*0.008*	*0.0285*	*0.077*
	50	0.1475*	*0.001*	*0.0599*	*0.002*	*0.0321*	*0.058*
	100	0.1472*	*0.001*	*0.0593*	*0.008*	*0.0329*	*0.066*
Urban area	2	0.1390*	0.001	0.0078	0.313	−0.0019	0.513
	5	0.1392*	**0.001**	0.0121	0.243	0.0036	0.430
	25	0.1390*	0.001	0.0169	0.161	0.0149	0.207
	50	0.1381	0.001	0.0148	0.208	0.0202	0.151
	100	0.1375	0.001	0.0128	0.248	0.0231	0.104
Open water	2	0.1394*	0.001	0.0219	0.132	−0.0173	0.836
	5	0.14010*	**0.001**	0.0211	0.113	−0.0084	0.681
	25	0.14008*	0.001	0.0204	0.125	0.0067	0.339
	50	0.1395*	0.001	0.0182	0.159	0.0118	0.257
	100	0.1390*	0.001	0.0167	0.194	0.0143	0.254
Special matrix	2	0.1387	0.001	−0.0063	0.628	0.0080	0.314
	5	0.1385	0.001	−0.0079	0.678	0.0123	0.242
	25	0.1378	0.001	−0.0100	0.707	0.0196	0.124
	50	0.1375	0.001	−0.0116	0.761	0.0224	0.099
	100	0.1373	0.001	−0.0123	0.769	0.0236	0.083
Wetland	2	0.1387	0.001	−0.0069	0.664	0.0077	0.338
	5	0.1387	0.001	−0.0045	0.586	0.0070	0.363
	25	0.1385	0.001	−0.0026	0.583	0.0085	0.308
	50	0.1385	0.001	−0.0026	0.564	0.0093	0.289
	100	0.1385	0.001	−0.0027	0.543	0.0098	0.278
Females (*n* = 35)							
Null		0.2373	0.001				
Forest	2	0.2290	0.001	−0.0628	0.921	0.0895	0.037
	5	0.2270	0.001	−0.0365	0.759	0.0798	0.053
	25	0.2261	0.001	−0.0360	0.776	0.0823	0.054
	50	0.2260	0.001	−0.0361	0.737	0.0825	0.045
	100	0.2260	0.001	−0.0361	0.776	0.0825	0.047
Grass field	2	0.2349	0.001	−0.0435	0.848	0.0557	0.082
	5	0.2285	0.001	−0.0459	0.857	0.0804	0.024
	25	0.2152	0.001	−0.0344	0.793	0.1079	0.004
	50	0.2145	0.001	−0.0298	0.759	0.1081	0.005
	100	0.2139	0.001	−0.0309	0.783	0.1095	0.005
Farmland	2	0.2475*	*0.001*	*0.0911*	*0.031*	*−0.0557*	*0.874*
	5	0.2601*	*0.001*	*0.1119*	*0.009*	*−0.0228*	*0.667*
	25	0.2626*	*0.001*	*0.1190*	*0.011*	*0.0283*	*0.277*
	50	0.26338*	*0.001*	*0.1210*	*0.008*	*0.0286*	*0.283*
	100	0.26339*	***0.001***	*0.1210*	*0.005*	*0.0286*	*0.260*
Urban area	2	0.2384*	0.001	0.0391	0.186	−0.0316	0.755
	5	0.2394*	0.001	0.0364	0.187	−0.0161	0.637
	25	0.2401*	0.001	0.0380	0.184	0.0049	0.430
	50	0.2402*	0.001	0.0389	0.182	0.0068	0.436
	100	0.2403*	**0.001**	0.0395	0.165	0.0075	0.433
Open water	2	0.2378*	0.001	0.0168	0.350	−0.0066	0.562
	5	0.2385*	**0.001**	0.0241	0.302	0.0027	0.462
	25	0.2381*	0.001	0.0298	0.221	0.0223	0.285
	50	0.2378*	0.001	0.0298	0.242	0.0254	0.282
	100	0.2377*	0.001	0.0299	0.249	0.0263	0.278
Special matrix	2	0.2374*	0.001	0.0323	0.253	−0.0317	0.747
	5	0.2376*	0.001	0.0353	0.247	−0.0333	0.750
	25	0.2380*	0.001	0.0375	0.176	−0.0324	0.753
	50	0.23816*	0.001	0.0365	0.219	−0.0301	0.736
	100	0.23822*	**0.001**	0.0373	0.196	−0.0307	0.731
Wetland	2	0.2374*	0.001	0.0363	0.227	−0.0359	0.766
	5	0.2375*	0.001	0.0366	0.200	−0.0356	0.783
	25	0.2379*	0.001	0.0457	0.163	−0.0426	0.824
	50	0.2380*	0.001	0.0468	0.132	−0.0428	0.808
	100	0.2381*	**0.001**	0.0473	0.169	−0.0431	0.837

Notes

Asterisks show Mantel *r* values which are higher than those of null model (the Euclidean distance). Bold numbers indicate the highest Mantel *r *of each model. Italic character indicates that the resistance values could act as the resistance for the species according to the concept Ruiz‐Gonzalez et al. (2014).

When we examined gene flow for pairs of males and pairs of females, we found that forest, farmland, urban area, and open water showed higher Mantel *r* values than the Euclidean distance for males, with the highest Mantel *r* value being 25 for forest and farmland, and 5 for both urban area and open water. However, only farmland was indicated to have a significant resistance influence on genetic distance by partial Mantel tests (*G × Dis|R_L_* and *G × R_L_|Dis*). For females, farmland, urban area, open water, special matrix, and wetland showed higher Mantel *r* values than the Euclidean distance, and the resistance value with highest Mantel *r* values of 100 for farmland, urban area, special matrix, and wetland, and 5 for open water. Partial Mantel tests (*G × Dis|R_L_* and *G × R_L_|Dis*) indicated a significant resistance influence on genetic distance only for farmland according to Ruiz‐Gonzalez et al. (2014).

We additionally performed simple (*G × *Σ*R_L_*) and partial (*G × *Σ*R_L_|Dis* and *G × Dis|*Σ*R_L_*) Mantel tests based on the combined resistance of land uses, in which the resistance values were 25 for farmland and urban use for all sample pairs. We accordingly found that the highest Mantel *r* value (0.0794, *p* = 0.001) was obtained using the simple Mantel test (*G × *Σ*R_L_*). The partial Mantel test with partialling out of Euclidean distance (*G × *Σ*R_L_|Dis*) also had a high Mantel *r* value (0.0403, *p* < 0.01), whereas *G × Dis|*Σ*R_L_* had a negative Mantel *r* value (0.0051, *p* > 0.05), and thus, we could conclude that the combined resistance values act as a resistance to the gene flow of this species.

### 3.4 The barrier function of land use on gene flow

Analysis of the correlation between genetic distance and the presence of candidate land use type using a simple Mantel test (*G × B_L_*) revealed that Mantel *r* values for farmland (0.0787) for all sample pairs and forest (0.2558) for female pairs were higher than those between the Euclidean distance and genetic distance (0.0687 and 0.2373, respectively), whereas no land use type showed higher a Mantel *r* value than the Euclidean distance for male pairs (Table 4). When we analyzed the barrier function of farmland area using partial Mantel tests to assess the relationship between genetic distance and the presence of farmland with partialling out of the Euclidean distance of the network (*G × B_L_|Dis*) and between genetic distance and the Euclidean distance with partialling out the presence of farmland (*G × Dis|B_L_*), we found that farmland could act as a genetic distance barrier (*G × B_L_|Dis*: 0.0399: *p* < 0.01, *G × Dis|B_L_*: 0.0176: *p* > 0.05), according to the concept of Ruiz‐Gonzalez et al. (2014). However, the value of Mantel *r* (0.0787) was smaller than that of the resistance model (0.0794 for *G × *Σ*R_L_*).

**Table 4:** Results of simple and partial Mantel test detecting landscape barrier of land use

Model of land use	G*BL		G*BL|Dis		G*Dis|BL	
	Mantel *r*	*p* value	Mantel *r*	*p* value	Mantel *r*	*p* value
All samples (*n* = 148)						
Null	0.0687	0.001				
Forest	0.0074	0.322	−0.0053	0.628	0.0700	0.001
Grass field	0.0238	0.042	−0.0181	0.948	0.0684	0.001
Farmland	0.0787*	*0.001*	*0.0399*	*0.004*	*0.0176*	*0.124*
Urban area	0.0576	0.002	0.0330	0.006	0.0519	0.002
Open water	0.0040	0.409	−0.0240	0.966	0.0740	0.001
Special matrix	0.0046	0.353	−0.0036	0.620	0.0701	0.001
Wetland	−0.0023	0.567	−0.0081	0.747	0.0706	0.001
Males (*n* = 87)						
Null	0.1388	0.001				
Forest	0.0983	0.001	0.0554	0.002	0.1128	0.001
Grass field	0.0463	0.015	−0.0092	0.673	0.1313	0.001
Farmland	0.1214	0.001	0.0618	0.002	0.0916	0.001
Urban area	0.0547	0.007	0.0232	0.119	0.1298	0.001
Open water	0.0429	0.015	0.0172	0.201	0.1332	0.001
Special matrix	−0.0073	0.682	−0.0137	0.776	0.1393	0.001
Wetland	0.0074	0.345	0.0061	0.334	0.1387	0.001
Females (*n* = 35)						
Null	0.2373	0.001				
Forest	0.2558*	0.001	0.1661	0.001	0.1346	0.004
Grass field	0.0493	0.111	−0.0523	0.89	0.2379	0.001
Farmland	0.1780	0.001	0.0680	0.072	0.1731	0.001
Urban area	0.0820	0.032	0.0188	0.322	0.2242	0.001
Open water	−0.0095	0.581	−0.0617	0.907	0.2446	0.001
Special matrix	0.0400	0.179	0.0033	0.469	0.2341	0.001
Wetland	0.0577	0.104	0.0322	0.233	0.2327	0.001

Notes

Asterisks show Mantel *r* values which are higher than those of null model (the Euclidean distance). Italic character indicates that the resistance values could act as the resistance for the species according to the concept Ruiz‐Gonzalez et al. (2014).

In contrast, for females, it appeared that forest does not act as genetic distance barrier (*G × B_L_|Dis*: 0.1661: *p* < 0.01, *G × Dis|B_L_*: 0.01346: *p* < 0.01), according to Ruiz‐Gonzalez et al. (2014).

## 4 DISCUSSION

Gradient concepts such as IBD are sometimes consistent with the genetic structure of continuous populations (Ohnishi, Kobayashi, Nagata, & Yamada, 2017; Pelletier et al., 2012). Although we also detected an IBD effect in the Asian black bear population investigated in the present study, Bayesian clustering analysis suggested a certain degree of genetic clustering. This prominent structuring could be a consequence of gene flow disruption caused by landscape heterogeneity. We determined which landscape factors influence the gene flow producing genetic clusters and concluded that they provided functional resistance to gene flow.

Cushman et al. (2013) have explained the IBD patterns determined by landscape heterogeneity based on behavioral ecology. Animal movement behavior is selected to maximize fitness with respect to resources, such as food, dens, and reproductive partners, and to minimize the risk of predation. Continuous uniform landscapes, by definition, have low heterogeneity, thereby inducing animals to select differential paths. In such landscapes, movement generally conforms to a random walk model, which gives rise to an IBD pattern and does not support IBR. In contrast, in landscapes with high heterogeneity and fragmented habitats, movement paths will be selected primarily to avoid unsuitable conditions. As a consequence, an IBR pattern in path selection will be observed.

In the present study, we confirmed these assumptions based on our analyses of different landscape features, namely, elevation and land use. The maximum difference and unevenness in elevation between individuals represent functional heterogeneities for the present population. Although we were unable to detect an effect of elevation per se in the black bear population we studied, elevation has been implicated in studies on American black bears (Cushman et al., 2006; Short Bull et al. 2011). The difference in the findings of the American black bears studies and those of the present study may be explained by differences in the elevational ranges in the respective study areas. The elevational ranges of the American black bears study areas are from <2,000 m to over 7,000 m, whereas, the highest mountain in the present study area reaches only 2,236 m. Both Asian and American black bears are omnivores, and the foraging diet of individuals is believed to be learnt from their mothers (Kitamura & Ohnishi, 2011; Mazur & Seher, 2008). Given that vegetation changes according to elevation, American black bears would tend to disperse to areas with an elevation similar to that of their birthplace to locate familiar foods. In contrast, there is little restriction in accessing vegetation for the bears in the present study area due to the relatively low elevation. Consequently, a change in elevation on the route of bear movements would not represent a resistance to gene flow. It has been suggested that elevation does not influence gene flow in American black bear populations in landscapes where the topography is relatively flat and elevation is not highly variable (Short Bull et al. 2011). However, the results of the present study indicate that a topographic influence can be detected in nonflat landscapes, even though the range of elevation is small, and we demonstrate that unevenness alone could provide functional resistance to gene flow.

Even though IBD was detected in the present study, we detected a resistance value of 25 for farmland and urban area for all sample pairs. By combining the models of these land use types, we succeeded in generating an IBR model that had the highest Mantel *r* values (0.0794). However, the single IBR model for farmland showed higher Mantel *r* values for male and female pairs (0.1485 for males and 0.2634 for females). All simple Mantel *r* values including Euclidean distance were higher for females than for males, and the resistance value for farmland was four times larger for females than for males. This tendency can probably be explained by differences in the dispersal patterns and social systems of males and females. Natal philopatry in female‐ and male‐biased dispersal have been suggested (Ohnishi & Osawa, 2014), and matrilineal site fidelity has also been detected in black bears (Kozakai et al., 2017). Additionally, a recent behavioral study also suggested that female tended to avoid farmland more than male (Takahata, Takii, & Izumiyama, 2017). An interesting point in this regard is that special matrix and wetland appear to show resistance to females, even though a partial Mantel test did not support the resistance of land use. We assume, however, that farmland has a resistance function because it represents an artificial land use, whereas special matrix and wetland are natural landscapes. These results tend to indicate that females are strongly dependent on forests and grass fields for their habitat. In contrast, these natural land uses did not show resistance to males, but we do not believe that these males are independent of forests and grass fields. The capacity of long‐distance dispersal and larger home ranges would enable males to circumvent these particular landscape types. Moreover, the difference in habitat preference would also cause these results because it is suggested that males select open area during summer but females avoid it during all seasons (Takahata et al., 2017).

Although the difference in the results of the Mantel tests between sexes was, as mentioned above, as predicted, our results for all sample pairs tended to be a little less clear‐cut. A previous study on the fisher *Pekania pennanti* indicated that sex‐biased dispersal results in different Mantel *r* values: females > all samples > males (Tucker et al., 2017). We also predicted that Mantel *r* values for all sample pairs are lower than those for female pairs but higher than those for male pairs; however, we observed that the Mantel *r* values for all pairs were actually lower than those for the pairs of each sex. The same tendency in the Mantel *r* values of IBD has been confirmed in a previously published study (Ohnishi, Yuasa, Morimitsu, & Oi, 2011), in which authors used hair samples collected in different areas using hair traps (−0.113 for females, −0.112 for males, and −0.099 for all sample pairs), even though they obtained negative Mantel *r* values because of relatedness as genetic distance. Therefore, we assume that this tendency could be a common characteristic of black bear populations in Japan. Accordingly, it may also be necessary to consider relationships among individuals not only in terms of sex‐biased dispersal patterns but also with respect to behavior pattern. Strong matrilineal relationships after natal dispersal is suggested in females (Kozakai et al., 2017) and there are assumed to be certain male–male relationships, although to date these have not been investigated. Furthermore, difference in habitat selection between sexes in each season would tend to cause lower IBD and IBR. For example, females select subalpine forests during spring and deciduous forests during summer, but males do not prefer to them during both seasons (Takahata et al., 2017). Accordingly, we propose that these male–male and female–male relationships should be studied combined with behavioral investigations.

On the basis of our current findings, we could reject the likelihood of IBB operating in the bear population we studied, which has also been indicated in the case of American black bears (Cushman et al., 2006). Whereas resistance can show degrees of variability, barriers tend to be absolute (i.e., 0 or 1). Such an immoderate habitat would be basically island surrounded by sea or lake for terrestrial animal. We can thus assume that barriers provide extremely strong resistance to gene flow. For example, Saitoh et al. (2001) and Ohnishi et al. (2007) revealed large genetic differentiation among the black bear populations fragmented by rivers in western Japan and concluded that the rivers constitute functional barriers to gene flow among fragmented populations. However, low frequency movements across rivers are also suggested (Ishibashi & Saitoh, 2004; Ohnishi et al., 2009). Nevertheless, given that residential areas and farmlands tend to concentrate along either side of river courses, the landscape in the vicinity of rivers would have strong functional resistance.

## CONFLICT OF INTEREST

None declared.

## AUTHOR CONTRIBUTIONS

N.O. performed design and DNA analyses, and wrote the manuscript; T.O. conducted landscape and statistical analyses; T.Y. and R.U. contributed samples; all authors contributed to the final versions of the manuscript.

## Data Availability

We submitted genotype data to the Dryad database on 12 July 2018. https://doi.org/10.5061/dryad.gn0qf16.
